# The praziquantel in preschoolers (PIP) trial: study protocol for a phase II PK/PD-driven randomised controlled trial of praziquantel in children under 4 years of age

**DOI:** 10.1186/s13063-021-05558-1

**Published:** 2021-09-06

**Authors:** Emily L. Webb, Andrew Edielu, Hannah W. Wu, Narcis B. Kabatereine, Edridah M. Tukahebwa, Alfred Mubangizi, Moses Adriko, Alison M. Elliott, William W. Hope, Patrice A. Mawa, Jennifer F. Friedman, Amaya L. Bustinduy

**Affiliations:** 1 MRC International Statistics and Epidemiology Group, London School of Hygiene and Tropical Medicine, London, UK; 2 MRC/UVRI and LSHTM Uganda Research Unit, Entebbe, Uganda; 3 Department of Clinical Research, London School of Hygiene and Tropical Medicine, London, UK; 4 Department of Pediatrics, Alpert Medical School of Brown University, Providence, RI USA; 5 Center for International Health Research, Lifespan Hospital, Providence, RI USA; 6 Vector Control Division, Ministry of Health, Kampala, Uganda; 7 Antimicrobial Pharmacodynamics and Therapeutics, University of Liverpool, Liverpool Health Partners, Liverpool, UK; 8 Royal Liverpool, Broadgreen University Hospital Trust, Liverpool Health Partners, Liverpool, UK; 9 Department of Immunology, Uganda Virus Research Institute, Entebbe, Uganda; 10 Department of Infection Biology, London School of Hygiene and Tropical Medicine, London, UK

**Keywords:** Praziquantel, Preschool, Children, Intestinal schistosomiasis, *Schistosoma mansoni*, *Schistosoma japonicum*

## Abstract

**Background:**

Over 200 million individuals worldwide are infected with *Schistosoma* species, with over half of infections occurring in children. Many children experience first infections early in life and this impacts their growth and development; however praziquantel (PZQ), the drug used worldwide for the treatment of schistosomiasis, only has regulatory approval among adults and children over the age of four, although it is frequently used “off label” in endemic settings. Furthermore, pharmacokinetic/pharmacodynamics (PK/PD) evidence suggests the standard PZQ dose of 40 mg/kg is insufficient in preschool-aged children (PSAC). Our goal is to understand the best approaches to optimising the treatment of PSAC with intestinal schistosomiasis.

**Methods:**

We will conduct a randomised, controlled phase II trial in a *Schistosoma mansoni* endemic region of Uganda and a *Schistosoma japonicum* endemic region of the Philippines. Six hundred children, 300 in each setting, aged 12–47 months with *Schistosoma* infection will be randomised in a 1:1:1:1 ratio to receive either (1) 40 mg/kg PZQ at baseline and placebo at 6 months, (2) 40 mg/kg PZQ at baseline and 40 mg/kg PZQ at 6 months, (3) 80 mg/kg PZQ at baseline and placebo at 6 months, or (4) 80 mg/kg PZQ at baseline and 80 mg/kg PZQ at 6 months. Following baseline treatment, children will be followed up for 12 months. The co-primary outcomes will be cure rate and egg reduction rate at 4 weeks. Secondary outcomes include drug efficacy assessed by novel antigenic endpoints at 4 weeks, actively collected adverse events and toxicity for 12 h post-treatment, morbidity and nutritional outcomes at 6 and 12 months, biomarkers of inflammation and environmental enteropathy and PZQ PK/PD parameters.

**Discussion:**

The trial will provide valuable information on the safety and efficacy of the 80 mg/kg PZQ dose in PSAC, and on the impact of six-monthly versus annual treatment, in this vulnerable age group.

**Trial registration:**

ClinicalTrials.gov NCT03640377. Registered on 21 Aug 2018.

**Supplementary Information:**

The online version contains supplementary material available at 10.1186/s13063-021-05558-1.

## Background

Schistosomiasis is a water-borne, disabling parasitic disease affecting over 200 million individuals globally and accounting for over 1.4 million disability-adjusted life years (DALYS) [1]. There are three main species of tissue-invasive parasitic trematodes of the genus *Schistosoma* (*S*.), responsible for causing schistosomiasis-related morbidity in different geographic areas: *S. mansoni* in sub-Saharan Africa (SSA) and South America, *S. haematobium* in SSA and *S. japonicum* in Asia [2]. A significant proportion of the global burden of schistosomiasis comprises key morbidities that disproportionally affect children [3]. Specifically, growth and development related disabilities attributed to schistosomiasis include undernutrition, linear growth stunting [4], anaemia [5] and cognitive impairment [6]. Importantly, young children are most vulnerable to these morbidities given this period coincides with peak velocities of somatic and brain growth, with the highest global prevalence of anaemia, linear growth stunting, decreased aerobic capacity [7] and undernutrition occurring in this age group worldwide [8, 9]. Despite this vulnerability and likely increased risk of morbidity due to schistosomiasis, preschool-aged children (PSAC) are excluded from preventive chemotherapy campaigns, which represent the primary approach for reducing infections in endemic regions [10, 11]. Few if any studies have evaluated the effect of treatment on these key indicators of early childhood well-being.

The persistent treatment gap among PSAC is partially driven by the fact that praziquantel (PZQ), the recommended treatment against all forms of schistosomiasis, has regulatory approval only for children aged 4 years and older [12]. National control strategies often focus on school-based treatment, with community treatment recommended only in highly endemic areas. The World Health Organization (WHO) recommends that PZQ at a dose of 40 mg/kg be used to treat schistosomiasis infection in PSAC [13]. However, this recommended dose relied predominantly on differences in cure and egg reduction rates at different doses from a relatively small number of studies. The 40-mg/kg dose was also based on extrapolating from the relatively few pharmacokinetic/pharmacodynamics (PK/PD) studies of healthy adults as well as adults with varying degrees of liver failure. Despite the known flaws of this approach, including evidence from quantitative pharmacological methods that an extrapolation approach does not achieve accurate paediatric dosing, this continues to define dosing regimens for children [14, 15]. This is of significant concern given the many differences in drug absorption, metabolism and distribution among children compared to adults [16]. Weight alone does not capture age-dependent nonlinearities in drug metabolism [14]. Specifically, body weight-normalised drug clearance in children exceeds that of adults for many drugs [16], suggesting children may require a higher dose per unit of bodyweight.

A recent PK/PD study comparing 40 mg/kg versus 60 mg/kg of PZQ among children aged 3–8 years living in a *S. mansoni* endemic region of Uganda found that the total PZQ area under the concentration time curve (AUC) was a significant predictor of both cure rate and egg reduction rate, while administered dose was not a significant predictor [17]. Subsequent modelling suggested that doses much higher than 40 mg/kg will be necessary to achieve cure rates of 85% as recommended by the WHO, particularly among younger children [17]. Specifically, it is likely that 80 mg/kg is necessary to achieve recommended cure rates.

Through the PZQ in preschoolers (PIP) trial, we will address the significant gaps with respect to our understanding of the best approaches to optimise treatment of PSAC with intestinal schistosomiasis including both optimal dose (40 vs 80 mg/kg) and frequency of dosing (once or twice annually).

## Methods

The PIP trial protocol is reported following Standard Protocol Items: Recommendations for Interventional Trials (SPIRIT) guidelines (Additional file 1) [18].

### Trial objectives

The primary objective of this trial is to compare parasitological cure and egg reduction rate of PZQ at a dose of 80 mg/kg versus PZQ at a dose of 40 mg/kg, among children aged 12–47 months. Secondary objectives include the following:

- To assess the safety and efficacy and PK/PD of PZQ administered at 80 mg/kg versus 40 mg/kg
- To evaluate the PK/PD of both PZQ enantiomers (R/S) given the concern that this has varied in studies of *S. mansoni*
- To expand PD endpoints for drug efficacy to include antigen tests (circulating anodic antigen; CAA) that accurately capture residual worm burden, and assess the impact of 80 mg/kg versus 40 mg/kg PZQ on these endpoints
- To assess the impact of 80 mg/kg PZQ versus 40 mg/kg PZQ and of annual versus biannual PZQ treatment on key measures of growth and morbidity
- To evaluate the mechanisms mediating the effect of varying PZQ treatment doses and frequencies on growth and schistosomiasis-related morbidity (nutritional status, anaemia)
- To evaluate the mechanistic role of environmental enteric dysfunction (EED) in the pathogenesis of schistosomiasis related morbidities
- To explore immune responses to *S. mansoni* infection and treatment
- To determine the prevalence and the pattern of schistosomiasis-related morbidity as determined by ultrasound scan

### Trial design

**Fig. 1:**
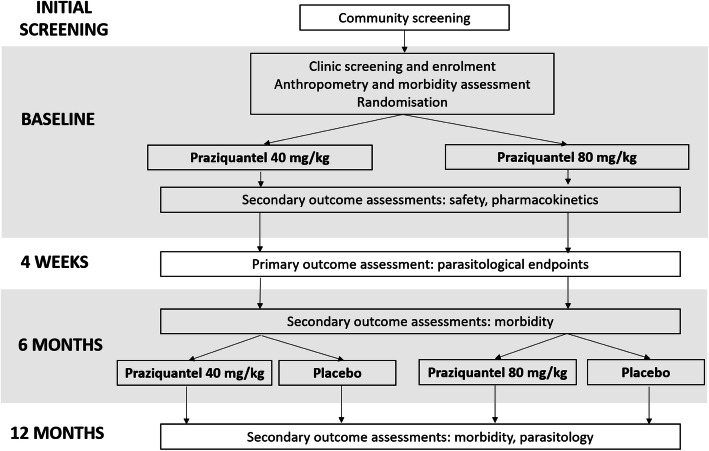
Schematic of study design

**Fig. 2:**
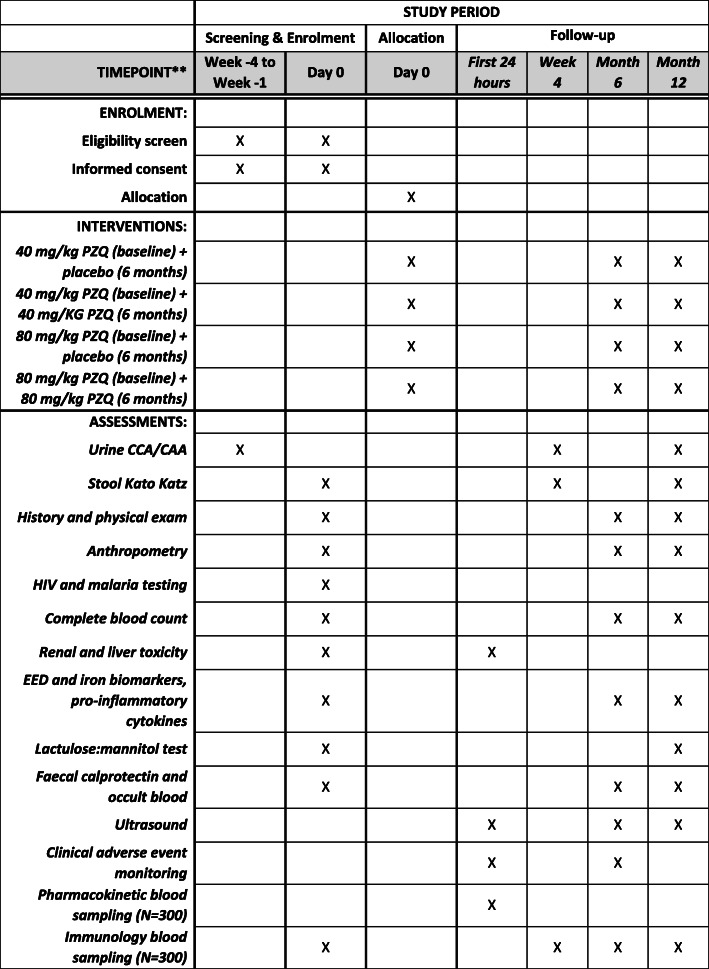
PIP trial schedule of enrolment, interventions and assessments

This will be a two-centre individually randomised quasi-double blinded placebo-controlled trial with four parallel arms (Fig. 1). Children aged 12–47 months will be enrolled and followed up at 4 weeks, 6 months and 12 months post-baseline. All participants will receive PZQ (at either 80 mg/kg or 40 mg/kg) at baseline. Half of the cohort will receive an additional PZQ dose (at the same dose as they were given at baseline) at 6 months and half will receive placebo at 6 months. Parasitological endpoints will be measured at 4 weeks, morbidity endpoints at 6 and 12 months. The schedule of participant enrolment, interventions and assessment is shown in Fig. 2.

### Trial settings

The trial will be conducted in a *S. mansoni* endemic setting in Uganda and a *S. japonicum* endemic setting in the Philippines. In Uganda, participants will be recruited from villages located on the shores of Lake Albert in Buliisa district in western Uganda. A recent study in these villages found *S. mansoni* prevalence of 57% by Kato-Katz among children aged 3–5 years [19]. The general health of children is poor, and there are high rates of undernutrition with approximately 38% stunted and 16% underweight [20]. The Uganda Ministry of Health Vector Control Division has a long-established field station, the Kabatereine Schistosomiasis Research Camp, located at Bugoigo village, which has recently been updated to accommodate the facilities necessary to conduct study procedures. In the Philippines, participants will be recruited from rice farming villages in Leyte district, with further study procedures conducted at the field laboratory in Palo, Leyte. In a community survey of these villages conducted in May 2016, *S. japonicum* infection prevalence among 1–4-year-olds was 42%. In addition, 67% of children were stunted (height-for-age *z*-score < − 2) and 15% were wasted (BMI-for-age *z*-score < − 2) [21]. In both study settings, the population size per village ranges from approximately 600 to 1900 individuals, and approximately 10% of the population are aged under 4 years. It is anticipated that participants will be recruited from approximately 15–20 villages across the two study settings to achieve the planned sample size.

### Screening and eligibility

Fishing and rice farming communities where schistosomiasis is endemic will be approached through community-wide information meetings. Prior to these, the village chiefs and leaders will be contacted to inform them about the project and obtain permission to hold meetings. These meetings will be announced publicly with flyers distributed by community mobilisers who will be in charge of contacting interested parents after the information sessions have taken place.

Screening will take place in two phases. The first phase will comprise circulating cathodic antigen (CCA) testing of urine samples [22, 23] from potential participants in the villages by field staff after informed consent for this initial screening activity has been obtained. Children with a positive CCA test will be considered positive for *Schistosoma* infection and eligible for potential enrolment. Parents will then be given three cups to provide stool samples collected on different days to ascertain egg-patent infection and quantify intensity of infection at baseline.

The second screening phase will take place at the field laboratories (in Bugoigo for Uganda, and Palo for the Philippines) to ensure other eligibility criteria are met prior to enrollment. Activities related to the second phase of screening and the main trial procedures will be reviewed and informed consent obtained (Supplemental Information). The second phase of screening will include an assessment of nutritional status, laboratory studies and physical examination and history to rule out conditions that might deem a child ineligible.

Inclusion criteria will be as follows:

- *S. mansoni* infection of any intensity as determined by Kato-Katz
- Otherwise healthy as determined by history and physical examination conducted by the study physician at the second stage screening
- Age 12–47 months inclusive
- Parental consent to participate

Exclusion criteria will be as follows:

- Parental inability to provide informed consent
- Significant disease/illness as determined by history or physical exam. This includes a severe acute illness or chronic disease as defined by greater than 3 months’ duration and significantly impacting a child’s daily activities.
- Grade 3 or higher laboratory abnormality of blood urea nitrogen (BUN), creatinine, bilirubin, white blood cell count, or platelet count will warrant exclusion. Grade 2 or higher abnormality of alanine aminotransferase (ALT) or aspartate aminotransferase (AST) will warrant exclusion. Children with severe anaemia as defined by haemoglobin less than 7.0 g/dl will be excluded.
- Severe wasting as defined by weight-for-height *z*-score < − 3
- Exposure to immuno-modulatory therapeutics such as steroids e.g. dexamethasone, prednisone and other medications such as chloroquine
- Confirmed diagnosis of ocular cysticercosis

### Information and consent

Information sheets will be provided to interested parents/guardians of potential participants. Information sheets will be available in English and the main local languages. Parents will be provided with an oral and written explanation of the study to ensure that information is accessible. The informed consent process for the first phase of screening will be conducted by good clinical practice (GCP) certified community workers. The informed consent process for the second phase of screening will be conducted by project staff trained in GCP and who speak the local language. Consent will also be obtained for use of stored samples. Witnessed consent will be available to parents or guardians who are non-literate.

### Withdrawal of participants

At all stages, it will be made clear to parents of study participants that they may voluntarily withdraw consent for their child’s participation in the study at any time, without penalty or loss of benefits, and without needing to provide any reason or explanation. The study team may also withdraw a participant from receiving the study product for any reason, but follow-up safety evaluations will continue to be conducted, if the parent agrees. Any child experiencing a severe allergic or anaphylactic reaction to any dose of PZQ given during the study will be withdrawn by the study team.

### Interventions

Participants will be randomised to receive one of the following four interventions:

Group 1

- At baseline, 40 mg/kg PZQ followed by placebo 3 h later
- At 6 months post-baseline, placebo followed by placebo 3 h later
  Group 2
- At baseline, 40 mg/kg PZQ followed by placebo 3 h later
- At 6 months post-baseline, 40 mg/kg PZQ followed by placebo 3 h later
  Group 3
- At baseline, 80 mg/kg PZQ split as 40 mg/kg and 40 mg/kg 3 h later
- At 6 months post-baseline, placebo followed by placebo 3 h later
  Group 4
- At baseline, 80 mg/kg PZQ split as 40 mg/kg and 40 mg/kg 3 h later
- At 6 months post-baseline, 80 mg/kg PZQ split as 40 mg/kg and 40 mg/kg 3 h later

### Randomisation and masking

Placebo consisting of tablets matched for size, shape and colour with the PZQ tablets, will be prepared at Tedor Pharma, Cumberland, RI, USA and shipped to independent pharmacies in Entebbe, Uganda and Manila, The Philippines. Prior to first enrolment, a randomisation list, stratified by study site, indicating a randomisation number and trial arm allocation, will be prepared by the trial statistician using a random number generator with randomly permuted block sizes. The randomisation list will be shared with the independent pharmacists, who will be otherwise uninvolved in the trial. In each country, the pharmacist will pre-fill two vials per child for each study visit’s two doses based on their randomisation group, i.e. two doses at baseline and two doses at 6 months. Vials will be labelled with the assigned study number, according to the randomisation list. Following enrolment, children will be sequentially assigned to the next study identification number. Following baseline anthropometry, blood and urine lactulose:mannitol sampling, study staff will open the first vial labelled with that randomisation number and dispense the study product contained in the vial. This will be done according to weight, with tablets crushed and given with juice. Sufficient tablets will be included in each vial to allow for the maximum expected weight of a participant and repeat dosing (once only) in the event of vomiting within one hour of dosing. This will be a quasi-double blind approach as we will not be able to fully mask taste for participants.

### Outcomes

The trial will have two co-primary outcomes: (1) cure rate and (2) egg reduction rate at 4 weeks after baseline treatment. Cure rate will be defined as the proportion of participants who are Kao-Katz negative on all samples at 4 weeks. Egg reduction rate will be defined as the percentage reduction in egg burden from baseline to 4 weeks post treatment as assessed by Kato-Katz.

The secondary outcomes of the trial will be as follows:

- Drug efficacy by novel antigenic endpoint (CCA and CAA) [24] at 4 weeks
- Adverse events in children for 12 h following PZQ treatment at baseline and at 6 months
- Bone marrow, renal and liver toxicity for 12 h following PZQ treatment at baseline
- Iron status, haemoglobin, growth and nutrition at 6 and 12 months
- Biomarkers of inflammation and environmental enteric dysfunction (EED): faecal calprotectin and occult blood [25], serum endotoxin, serum endotoxin core antibody and pro-inflammatory cytokines at 6 and 12 months; urine lactulose:mannitol ratio at 12 months
- The PZQ PK/PD parameters including AUC for 12 h following baseline dosing, among a random sample of half the children
- Immune responses to *S. mansoni* infection and treatment at 4 weeks, 6 months and 12 months, among a random sample of half the children
- Prevalence and the pattern of schistosomiasis-related morbidity as determined by ultrasound scan at 6 and 12 months.

### Study procedures and baseline assessments: enrolment visit

Children meeting eligibility criteria will be admitted to the Bugoigo and Palo field stations for a period of up to 24 h for the enrolment visit. This visit will comprise baseline (pre-intervention) assessment of morbidity, anthropometry and nutrition outcome measures, administration of the baseline intervention, and PK/PD (for a random sample of half the children), adverse event and safety data collection following baseline intervention. Stool samples provided at screening will be used to determine baseline *Schistosoma* egg burden by microscopy. Faecal calprotectin and occult blood will be performed at the baseline visit. Blood samples (5 ml) will be taken for baseline assessment of complete blood count, and renal and liver function, with serum used for assessment of morbidity (iron status, EED biomarkers, measures of inflammation including pro-inflammatory cytokines and C-reactive protein (CRP)). A small blood drawing cannula will be placed to minimise the number of times children have a needle inserted. Rapid HIV and malaria testing will be administered following standard protocols.

A lactulose:mannitol test [26] will be administered to test gut permeability and absorptive capacity: following a 30-min fast (breast feeding will continue), lactulose-mannitol solution containing 250 mg/ml lactulose and 50 mg/ml mannitol in purified water will be given to study participants orally at a dose of 2 ml/kg up to a maximum of 20 ml. A urine bag will be strapped and urine will be collected for 2 h afterwards. Once collected, an aliquot of 2 ml urine will be stored between 2 and 8 °C having added 2 drops of chlorhexidine.

Following blood collection and completion of the urine lactulose:mannitol test, a meal of local foods will be given, and the study interventions will be dispensed as described under “Randomisation and masking”. After receiving the first study intervention, all children will be clinically monitored for 12 h and any adverse events will be recorded, with supportive treatment given if necessary. Data on anticipated adverse events (headache, malaise, drowsiness, abdominal pain, nausea, vomiting, shortness of breath, dizziness, rash, urticaria, fever) will be collected actively, while any unanticipated adverse events will also be captured as part of the clinical monitoring process. Bone marrow, renal and liver toxicity will be assessed in a final blood collection (3 ml) at 12 h post dosing. After administration of the second dose of the study product, children will undergo ultrasound scan to determine baseline presence or absence of any schistosomiasis-related liver pathology, with the Niamey Protocol used to grade severity of disease [27].

Participants will be randomly divided into two equally sized groups: one group (*N* = 150 at each site) will undergo additional blood sampling for PK assessment (four blood draws total over a 12-h period using the indwelling cannula, maximum total blood volume 5 ml) while the other group (*N* = 150 in each setting) will have 7 ml blood taken for assessment of immune responses to *Schistosoma* infection and treatment. At discharge, any child found to be infected with soil-transmitted helminths at enrolment will be treated with albendazole.

### Study procedures and outcome assessments: 4-week visit

The primary outcomes will be assessed at this visit, which will be conducted 4 weeks after the enrolment visit. Field workers will visit participants in their villages and collect stool samples on at least two consecutive days for assessment of *Schistosoma* egg burden by Kato-Katz. They will also collect urine samples for measurement of CCA and CAA. Children who were randomly selected at baseline for the assessment of immune responses to *Schistosoma* infection and treatment will have a repeat blood sample (7 ml) taken for these assessments.

### Study procedures and outcome assessments: 6-month visit

The 6-month visit will last approximately 3 h, will be done at the field station and will comprise assessment of morbidity, anthropometry and nutrition outcome measures, and administration of the 6-month PZQ or placebo intervention. Before PZQ/placebo administration, blood samples (5 ml) will be taken for outcome assessment of complete blood count, with serum used for assessment of morbidity (iron status, EED biomarkers, measures of inflammation), and stool samples will be collected for measurement of calprotectin and faecal occult blood. Children will be weighed and measured, a clinical history will be taken, and an ultrasound scan will be performed to determine possible changes compared to baseline morbidity patterns. Children who were randomly selected at baseline for the assessment of immune responses to *Schistosoma* infection and treatment will have a repeat blood sample (7 ml) taken for these assessments. Following a meal of local foods, children will receive PZQ/placebo according to the randomisation allocation, followed by clinical monitoring for post-treatment adverse events for a 3-h period, as for the baseline visit, anticipated adverse events will be actively enquired after and unanticipated adverse events will also be captured if reported.

### Study procedures and outcome assessments: 12-month visit

The final study visit will take place at the field station, 12 months after enrolment, and will comprise final assessment of morbidity, anthropometry and nutrition outcome measures. Children will be weighed and measured, and, for those children whose 6-month ultrasound scan did not demonstrate resolution of any pathology detected at baseline, a further ultrasound scan will be done. Blood samples (5 ml) will be taken for outcome assessment of complete blood count, with serum extracted for assessment of morbidity (iron status, EED biomarkers, pro-inflammatory cytokines). Urine samples will be collected for CAA/CCA. The lactulose:mannitol test will be repeated using the same procedures as described under the enrolment visit. Prior to the visit, field workers will collect at least two stool samples on consecutive days from study participants for Kato-Katz and for measurement of calprotectin and faecal occult blood. Children who are still infected with schistosomiasis will be treated with 40 mg/kg PZQ, and children infected with soil-transmitted helminths will receive albendazole treatment. Children who were randomly selected at baseline for the assessment of immune responses to *Schistosoma* infection and treatment will have a repeat blood sample (7 ml) taken for these assessments.

### Sample storage and shipment

Samples will be centrifuged and aliquoted into aliquots at each site and transported from Lake Albert to the MRC/UVRI and LSHTM Uganda Research Unit in Entebbe by road and from Leyte to the Research Institute for Tropical Medicine in Manilla by air, for storage at − 80 °C in dedicated clinical storage facilities. Further shipments to the USA (serum for markers of EED and iron status, urine for lactulose-mannitol) and to Liverpool (serum for pharmacokinetics) for these planned trial evaluations will be done. Subjects will be asked for permission to keep any samples remaining after the planned trial evaluations, for possible use in future research studies. These residual clinical samples will be stored indefinitely at the clinical storage facilities. Each sample will be labelled only with a barcode and a unique tracking number to protect subject confidentiality.

### Data management

Socio-demographic information and clinical and laboratory measurements will be recorded and managed using REDCap (Research Electronic Data Capture) tools [28, 29], with paper-based forms as back-up. All data will be recorded under a unique study ID number. When paper forms must be used, data will be double entered in a study-specific database, with standard checks for discrepancies. All data for analysis will be de-identified and stored on a secure and password-protected server, with access limited to essential research personnel.

### Sample size

We plan to recruit 600 children into the trial, 300 in Uganda and 300 in the Philippines. The first co-primary outcome will compare cure rate at 4 weeks in the 300 children who received 80 mg/kg PZQ at enrolment compared to the 300 children who received 40 mg/kg PZQ at enrolment. Data from Uganda estimate cure rates for *S. mansoni* infected 3–9 years olds of 70% and 82% for 40 mg/kg and 60 mg/kg PZQ, respectively [17]. Using a two-sided 5% significance level, approximately 173 children per trial arm will be required for 80% power and 232 per arm (*N* = 464) for 90% power to detect a difference in cure rate of 70% versus 82% at 4 weeks between the trial arms. Allowing for loss to follow-up of 7%, a total of approximately 500 children will be required. A sample size of 600 will allow us adequate power to examine sub-groups such as setting/*Schistosoma* species, younger (12–30 months) versus older (30–47 months) children, particularly given that cure rates may be lower in younger children and the effect size is likely to be larger based on a higher dose (80 mg/kg).

### Statistical analysis

A CONSORT trial flow chart will be constructed, showing the number of children screened, enrolled and randomised, together with reasons for exclusion at each stage. Baseline characteristics of trial participants will be summarised by trial arm. Numbers and proportions will be reported for categorical characteristics. Means, standard deviations, medians and ranges will be used for continuous characteristics. All trial analyses will be done using the intention-to-treat population, i.e. all children will be included as randomised, regardless of whether or not they received the allocated treatment. Children will contribute to 4-week outcomes if they are assessed within 1 week of the 4-week time point. Children will contribute to 6- and 12-month outcomes if they are assessed within 2 weeks of these time points. Loss to follow-up is expected to be low at 4 weeks since participants will be visited at their homes; therefore, only children for whom outcome data are available will be included in analyses (complete case analysis), with no imputation of missing outcome data planned. We will minimise losses to follow-up for later time points through continued engagement with the participants and their communities; field workers will remind participants of their next follow-up visit and will help with arranging transportation.

The co-primary outcome of cure rate will be compared between trial arms using a chi-squared test statistic. The difference in proportions cured and a corresponding 95% confidence interval (CI) will be calculated. For the co-primary outcome of egg reduction rate, mean egg reduction rate will be compared between trial arms using a *t* test, with the difference in mean egg reduction rate and its 95% confidence interval calculated. Schistosomal (CCA and CAA) antigenic egg clearance outcomes will be analysed using the same approach as for cure rate.

Prevalence of adverse events (both any and separately for types of event that occur with overall prevalence > 5%) will be compared between trial arms using chi-squared tests. Rare adverse events will be tabulated by trial arm but no formal statistical comparisons done. Severity of adverse events will also be tabulated, by trial arm. Both actively collected information on anticipated adverse events, and information on spontaneously-captured unanticipated adverse events will be reported. For the secondary outcomes assessed at 6 and 12 months (anthropometry and anaemia), continuous outcomes will be compared between trial arms using linear regression, including the corresponding baseline measure as a covariate. Binary outcomes assessed at 6 and 12 months will be compared between trial arms using binary/logistic regression, including the corresponding baseline measure as a covariate. This will be done in order to improve the precision of the treatment effect estimate.

Due to the large sample size, no imbalance is expected between trial arms. Therefore, no further adjustment for baseline characteristics is planned for any outcome, unless there are significant differences demonstrated at baseline for key potential confounders. We pre-specify three key potential effect modifiers to be examined. These include site (Uganda versus the Philippines), age (12–30 months versus 31–47 months) and baseline intensity of infection (low versus moderate/high). We will examine whether cure rate and egg reduction rate, as well as key measures of morbidity, differ by these sub-groups. Effect modification will be tested by fitting interaction terms in regression models. Full details of all planned analyses, including shell tables, will be included in a formal statistical analysis plan, to be finalised and reviewed before database lock.

### Trial management and data monitoring

A trial steering committee (TSC) and independent data safety and monitoring board (DSMB) have been established. The TSC comprises key study investigators and independent researchers and stakeholders with expertise in schistosomiasis, paediatrics, immunology and clinical trials in low- and middle-income countries. The DSMB membership comprises expertise in clinical trials, schistosomiasis, paediatrics, and statistics. An independent clinician with expertise in clinical trials has been appointed as an independent safety monitor (ISM), and will review unblinded serious adverse events (SAEs) and adverse events (AEs) for relatedness to the study intervention.

The DSMB will provide real-time safety oversight and may recommend that the investigators place the trial on hold if deemed necessary. They will have access to unblinded data upon request. They will be notified within 7 days of the investigators’ being aware of the occurrence of any SAE or any severe AE related to the trial interventions, or if any of the specified halting rules are met:

- ≥2 SAEs in the first 20 children associated with the study drug
- ≥5 SAES per 100 children associated with the study drug
- ≥14 subjects per 100 enrolled experiencing a clinical AE, that is not listed as expected with treatment, and that occurs within 2 days after dosing

The DSMB will also provide periodic review of safety and protocol fidelity and will review the results of an interim analysis to be performed on the primary outcomes when approximately 20% of children have been randomised and have accrued data on the primary outcomes. The trial will be monitored by both internal and external monitors according to a pre-defined monitoring plan which will include a site initiation visit, monitoring visits at least annually and a close-out visit. The monitors will assess patient safety, data integrity and adherence to the protocol and to Good Clinical Research Practice procedures.

If it is determined by the site principal investigator or project leader that an injury occurred to a subject as a direct result of participation in the trial, then immediate medical treatment will be provided by the participating site, with referrals to appropriate health care facilities provided if necessary. The trial also has insurance in place to pay for any unexpected, significant adverse events that are deemed to be due to the study drug.

### Dissemination activities

Study findings will be shared with the local community through community meetings, and with the wider scientific community through open access peer-reviewed publications, and presentations at local, national and international conferences. A website describing the PIP trial has been set up and will be used to share updates on trial progress and for dissemination of findings [30].

## Discussion

The PIP trial will allow us to evaluate optimal treatment dosing (80 mg/kg versus 40 mg/kg) and optimal intervals (6 versus 12 months) for intestinal schistosomiasis. We will assess the impact of these different treatment strategies on key indicators of morbidity that have been implicated in schistosomiasis, yet never previously studied among children under the age of four. Demonstrating efficacy with respect to morbidity will support the prioritization of treatment for this vulnerable age group and will significantly contribute to the impetus to provide treatment, even in resource-constrained settings.

One strength of the study design is the assessment of different doses of PZQ for the two primary species of intestinal schistosomiasis, *S. mansoni* and *S. japonicum*. Historically, PZQ dosing for *S. japonicum* treatment had been 60 mg/kg, higher than that for *S. mansoni* and *S. haematobium*, based on early efficacy and cure rate studies [31]. For over three decades, a higher dose of 60 mg/kg was used for *S. japonicum*. Recently, the WHO changed dosing recommendations to 40 mg/kg for all species in response to studies demonstrating similar egg reduction rates and cure rates at 40 and 60 mg/kg, however studies in *S. japonicum* endemic areas were under-represented [32] and no studies of children under four have evaluated this. In addition, no studies have assessed PK/PD in the context of *S. japonicum* in children.

Another strength is an examination of the impact of more frequent dosing on key morbidity outcomes. Children in this age group experience high prevalence of anaemia, undernutrition, and linear growth faltering. In areas of high endemicity, they may be rapidly re-infected, potentially at high intensity. Studies suggest that even low infections cause morbidity, particularly anaemia, such that more frequent treatment of infections may reduce this risk [33–35]. Furthermore, higher intensity infections have been shown to increase the risk for morbidity such that more frequent dosing in this vulnerable age group may have a more significant impact on morbidity if the intensity of infection is attenuated [36]. In addition, we will capture sophisticated markers of morbidity, including inflammatory and EED markers. This will allow us to better understand mechanisms of morbidity and immune responses to infection in PSAC, and how they are modified by treatment. Finally, we will be able to investigate the relationship between EED, which is known to affect absorptive capacity in the gut, and PK/PD markers to understand how EED may affect the absorption of PZQ.

Initial screening activities for the trial commenced in February 2020; however, following the emergence of the COVID-19 pandemic and subsequent national lockdowns, study activities were delayed for approximately 1 year; no participants had been randomised at the time the trial activities were paused. Screening and enrolment re-commenced in Uganda in March 2021 and is still pending in the Philippines. Study limitations include the use of large tablets that need to be crushed to be given to this age group. Since one of the main goals of the study was to inform dosing, a well-characterised dosage form and vehicle are needed to assess this. It is likely that these data can be extrapolated to inform dosing if other formulations such as liquid or oral dispersible tablets are approved. In addition, the use of cut and crushed tablets leads to imprecise dosing which may make interpretation of PK/PD data difficult. We will minimise this risk by using pharmacy grade pill cutters and crushers that deliver the dose into a small container for dispersing.

## Conclusion

This trial will inform optimal dosing and dosing frequencies for PZQ in this under-studied age group. It will also quantify and elucidate mechanisms through which schistosomiasis contributes to linear growth stunting, anaemia and undernutrition. It is hoped that this will inform expansion of mass drug administration programs to include this vulnerable age group.

## Trial status

The current PIP trial protocol is version 3.5 (July 2020). Trial recruitment is expected to commence in April 2021. We anticipate that recruitment will be completed in approximately 24 months and that the final follow-up of an enrolled participant will take place approximately 36 months after the first participant is enrolled.

## Supplementary Information

**Additional file 1.** SPIRIT Checklist.

**Additional file 2.** Supplemental Material: PIP Information and Consent Sheet.

## Data Availability

Data from the trial will be made available to interested investigators following IRB approval to provide these de-identified data. Specifically, after the research data set has been cleaned, finalised and all identifiers removed, the PIs will provide timely release and sharing of the final research data for use by other researchers. In addition, this study will generate samples collected from young children. Upon discussion with the Principal Investigators and based on the availability of samples, residual stored samples may be shared following IRB approval to provide these de-identified samples to interested researchers. Ethical approval for this will be obtained from participants.

## Abbreviations

AE: Adverse event
ALT: Alanine aminotransferase
AST: Aspartate aminotransferase
CAA: Circulating anodic antigen
CCA: Circulating cathodic antigen
CI: Confidence interval
CONSORT: Consolidated Standards of Reporting Trials
CRF: Case report form
DSMB: Data and Safety Monitoring Board
EED: Environmental enteric dysfunction
FDA: Food and Drug Administration
GCP: Good Clinical Practice
HIV: Human immunodeficiency virus
IQR: Interquartile range
ISM: Independent safety monitor
MRC: Medical Research Council
NIH: National Institutes of Health
PD: Pharmacodynamics
PIP: Praziquantel in preschoolers
PK: Pharmacokinetics
PZQ: Praziquantel
PSAC: Preschool-aged children
SAE: Serious adverse event
*S.*: *Schistosoma* genus
*S. mansoni*: *Schistosoma mansoni*
*S. japonicum*: *Schistosoma japonicum*
SOP: Standard operating procedure
SPIRIT: Standard Protocol Items: Recommendations for Interventional Trials
SSA: Sub-Saharan Africa
TSC: Trial Steering Committee
UK: United Kingdom
US: United States
UVRI: Uganda Virus Research Institute
VCD: Vector Control Division
WHO: World Health Organization
