# Supplementary material for: The praziquantel in preschoolers (PIP) trial: study protocol for a phase II PK/PD-driven randomised controlled trial of praziquantel in children under 4 years of age
Source: Trials. 2021 Sep 6;22:601. doi: 10.1186/s13063-021-05558-1 (PMC8419815; doi:10.1186/s13063-021-05558-1)
Supplement: Supplementary file 2 — Additional file 2. Supplemental Material: PIP Information and Consent Sheet. [file 13063_2021_5558_MOESM2_ESM.docx]

**Supplemental Material: PIP Information and Consent Sheet**

1. **Study Title:** *Praziquantel for children under four: A Phase II PK/PD driven dose finding trial (PIP trial)*
2. **Principal Investigators:** Dr. Amaya Bustinduy (LSHTM); Dr. Jennifer Friedman **(**Lifespan Hospital); Patrice Mawa (MRC/UVRI & LSHTM)
3. **Sponsor:** US NIH/NICHD
4. **Introduction:**

*This study is being conducted by a team from the Medical Research Council/Uganda Virus Research Institute and LSHTM Uganda Research Unit in collaboration with the London School of Hygiene &Tropical Medicine in the UK and Rhode Island Hospital in the US. This form will tell you about the research, to help you decide if you would like your child to take part in the study. Joining the study is entirely up to you and you will not lose any benefits or rights that you would normally enjoy for not taking part in this study. The study staff will talk with you about this information and answer any questions that you may have. Please read or listen carefully as it’s read to you and take as much time as you need. Please feel free to talk to others about this study if you wish.*

1. **What is the Purpose of the study?**

*Schistosomiasis (bilharzia) is a parasite infection which is very common around Lake Albert. Almost everyone who lives near the lake has bilharzia. Praziquantel is the medicine used to treat bilharzia, and is given to school children in the villages around the lake. However, nobody is sure about the best way of treating pre-school aged children with bilharzia. The purpose of this study is to find the a) best dose of medicine (praziquantel) to use to treat young children with schistosomiasis (bilharzia) and b) best frequency of treatment (every six or every 12 months) to decrease symptoms. One of the doses we will examine in this study is the same dose (40 mg of medicine for every 1 kilogram of weight) given at schools in your village for people over the age of four. The other dose (80 mg of medicine for every 1 kilogram of weight) is a new dose that we are studying because a previous study in Lake Albert in children 3-8 years of age found that 60 mg of medicine for every 1 kilogram of weight was not enough to treat bilharzia. Because 80 mg/kg is a bigger dose we will split the dose into two separate doses spread over 3 hours.*

1. ***Does my child qualify to participate in the study?***

*For your child to participate in this study, they should be between aged 12 – 47 months. In addition, we shall take blood from your child to ensure that their liver and kidneys are working properly so that the bad effects of the drug are not increased. Also ensure that your child has not taken chloroquine, dexamethasone, hydrocortisone, prednisolone in the last one month or grape juice (packed juice, usually dark in color). Your child’s nutritional state will also be assessed as well as presence or a certain worm which affects the eye.*

1. **Why have I been asked to take part?**

*You have been asked to take part because you live in a village where there is a lot of bilharzia, and your child has been found to have the infection. It is important to know what the best way of treating bilharzia is among young children.*

1. **Does my child have to take part?**

*No, participating in the study is voluntary*

1. **What will happen to me if I take part?**

*If you choose to allow your child to participate, we will ask that your child and a caregiver come to the Bugoigo Research Camp. The first time, you and your child will need to stay for about 12 hours to monitor your child and complete all of the necessary procedures. You and your child will be provided with a place to sleep in the Camp. Your child will first have a health check. If your child is not excluded based on any medical problem or laboratory results, we will be able to include them.*

1. *We will conduct measurements of your child’s body size (weight, height)*
2. *We will place a small catheter for blood drawing so we only have to “prick” your child once*
3. *We will collect a small volume of blood (about one teaspoon or 5 ml) to measure your child’s hemoglobin for anemia and other tests for your body’s response to infection (“cytokines”).*
4. *We will give them a sugar liquid to drink to measure how well their gut absorbs nutrients. We will collect urine that your child makes for two hours. This will be done by placing a small plastic bag over your child’s genital area.*
5. *We will conduct an ultrasound evaluation of your child’s abdomen. To do this we will put a small probe outside their abdomen to see a “picture” of their liver and spleen.*
6. *We will randomize your child to receive one of two different doses of Praziquantel in crushed tablets with juice for the treatment of their bilharzia. Randomization means that you are put into a group by chance, like flipping a coin. Your child will be provided with a meal before treatment. Your child will receive either 40 mg per 1 kg followed by a placebo (medicine with no activity) 3 hours later or 40 mg per 1 kg and repeated 40 mg per 1 kg 3 hours later for a total of 80 mg per 1 kg.*
7. *We will ask you to report any symptoms your child may have for 3-4 hours after treatment.*
8. *Half of the children will have one more blood draw (5 ml or one teaspoon more) for an immunology sub-study to understand how your child’s body fights bilharzia. The other half will have 4 more smaller blood draws (about 1-2 ml each for total of about one and a half teaspoons) for studies of how the drug for bilharzia is works in the body against the worm.*

***Summary of visit one:*** *I understand that my child will come to Buguigo camp, be examined and measured, have an ultrasound study of he/her belly, be treated for schistosomiasis with a “standard” dose or higher dose, have blood drawn, and sleep over. The whole visit will be about 20 hours.*

**Four weeks after treatment** the following activities will take place:

a*.*  *We will visit you at home collect two stool samples and one urine sample and see if your child still has schistosomiasis.*

*b. If your child was in the immunology sub-study group, we will ask you to come to the Bugoigo Research Camp for a blood draw of about 5 ml or 1 teaspoon.*

***Six months after treatment*** *the following activities will take place****:***

*a.*  *We will ask you to return to Bugoigo camp with your child for about 5-6 hours. We will give your child either the same dose of praziquantel they received at the beginning of the study or a placebo. Whether your child receives praziquantel or placebo will again be decided at random, e.g. like flipping a coin.*

*b. We will conduct the same measurements of your child’s body size (weight, height) and other urine studies as we did at the first visit.*

*c. We will collect a small volume of blood (about one teaspoon or 5 ml) to measure your child’s hemoglobin for anemia and other tests for your body’s response to infection (“cytokines”).*

*d. We will repeat the ultrasound study of your child’s abdomen*

*d. If your child was in the immunology sub-study group we will take an additional 5 ml or 1 teaspoon of blood*

***Summary of visit two in six months:*** *I understand that my child will come to Buguigo camp, be examined and measured, have an ultrasound study of he/her belly be treated for schistosomiasis or be given placebo,, and have blood drawn. The whole visit will be about 6 hours.*

**One year after the initial treatment** the following activities will take place**:**

1. *We will ask your child to provide a urine sample and two stool samples to test your child for schistosomiasis.*
2. *We will ask you to return to the Bugoigo camp with your child for about 5-6 hours.*
3. *We will conduct the same measurements, provide sugar liquid and collect blood (1 teaspoon or 5 ml) and urine as before.*
4. *We will repeat the ultrasound of your child’s abdomen*
5. *If your child is infected with schistosomiasis at this time, we will treat him/her with the same dose(s) as before.*
6. *We shall take another sample of blood (5 – 7ml) to help us better understand how your child reacted to the infection.*

***Summary of visit three in 12months:*** *I understand that my child will come to Buguigo camp, be examined and measured, have an ultrasound study of he/her belly, be treated for schistosomiasis if he/she is infected, and have blood drawn. The whole visit will be about 6 hours.*

1. **What are the possible risks and inconveniences?**

*Your child may feel a little uncomfortable with the blood draw. He/she may also have side effects from the medicine but there will be a doctor to monitor these symptoms. These symptoms include possible stomach pain, headache, dizziness, nausea, or vomiting.*

1. **What are the possible benefits?**

*Your child will receive treatment for schistosomiasis* *regardless of the treatment arm that he/she gets allocated.* *They will also be tested for malaria and other worms and receive treatment for these if they have them.*

1. **How many participants will be in this study?**

*Approximately 600 children aged 1-4 years old.*

1. **How long is this study?**

*The study will run for 2 years.*

1. **Can I change my mind about participating in the study?**

*Yes. You can withdraw from the study at any time without any consequence to you. You will not lose any legal rights because of your withdrawal from this study.*

1. **Can I be removed from the study while the study is ongoing?**

*The study team can also decide to stop you from participating in this study if, among other reasons, you do not attend the study visits, or if the sponsor of the study decides for any reason that it should not continue.*

1. **What will happen to information collected about me?**

*All information collected about your child will be kept private, kept in a secure place and will be accessed by only designated staff. Data may be sent to other study staff in the US or the UK but this will be anonymized, so that you and your child cannot be recognized. Your personal identifying details stored at LSHTM will be destroyed within 10 years of the end of the study.*

1. **What happens to the results of the study?**

*You will be notified when we know the results of the study. The study results will also be published in a medical journal so that other doctors can learn from them. Your personal information will not be included in the study report*

1. **What other choices do I have besides participating in this study?**

*Instead of participating in this research study, you may continue accessing care through the government health care.*

1. **Will it cost you anything to take part in this study?**

*There is no cost to you or your child for the study visits, examinations, or blood tests. Medical treatments that you need will be offered to you for free. If they are not available at the Bugoigo camp, you will be referred to a local public health facility for treatment and care. You will get a transport refund amounting to UGX. 20,000 every time you are invited to and attend Bugoigo camp.*

1. **Will you be paid to take part in this study?** *No*
2. **What will happen if I get sick or injured as a result of participation in this study?**

*We do not anticipate any injury as a result of participation this research study. The study team may provide care and treatment for concurrent illnesses not associated with the research study. If your child becomes ill or injured as a result of participation in this study, please inform the study staff immediately and they will provide treatment and pay costs.*

1. **What will happen to samples and data collected from this study?**

*All information collected about your child will be kept private, kept in a secure place and will be accessed by only designated staff. Data may be sent to other study staff in the US or the UK but this will be anonymized, so that you and your child cannot be recognized. Your personal identifying details stored at LSHTM will be destroyed within 10 years of the end of the study. At the end of the project, the data may be made available to other researchers worldwide for research and to improve medical knowledge and patient care. Your personal information will not be included and there is no way that you can be identified.*

1. **Who has allowed this research to take place?**

*This study was first reviewed by the Unit’s Scientific Committee prior to submission to an independent Research ethics committee (UVRI-REC) and Uganda National Council for Science and Technology (UNCST) and eventually National Drug Authority (NDA) who have looked carefully at this work and agreed that the research is important, it will be conducted properly and participants’ safety and rights have been respected.*

1. **Who should you call if you have questions regarding this study or if you get an injury?**

*If you have any questions about this study or a study-related injury, please contact Dr. Mawa the Ugandan principal investigator of this study on telephone number or the study coordinator of this study on* +**xx or +xx.**

*If you have any questions about your rights as a research participant, please contact: Mr. Tom Lutalo chairman of the UVRI-REC on telephone number xx, Entebbe (Uganda).*
